# The Relationship Between Leptin Receptor Expression and Endometrial Carcinoma; A Case-Control Study

**DOI:** 10.30699/ijp.2023.1972584.3017

**Published:** 2023-06-20

**Authors:** Mojtaba Sehhat, Zahra Moshfegh Arani, Niyusha Lajevardi Hosseini, Malek Reiesifar, Fahimeh Abdi Abyaneh, Zarichehr Vakili

**Affiliations:** 1 *Department of Social Medicine, School of Medicine, Kashan University of Medical Sciences, Kashan, Iran*; 2 *Department of Pathology, School of Medicine, Kashan University of Medical Sciences, Kashan, Iran*; 3 *School of Medicine, Kashan University of Medical Sciences, Kashan, Iran*; 4 *Surgical Pathologist, Isfahan, Iran*; 5 *Department of Pathology, Shahid Beheshti Hospital, Kashan University of Medical Sciences, Kashan, Iran*

**Keywords:** Endometrial carcinoma, Immunohistochemistry, Leptin receptor expression

## Abstract

**Background & Objective:**

Endometrial carcinoma is one of the most common malignancies in women in developed countries and the four^th^ malignancy in Iranian women. Therefore, the identification of its causative factors is essential for the prevention, diagnosis, and treatment. This study was aimed to compare the leptin receptor (Ob-R) expression in the endometrial carcinoma cases and non-carcinoma samples.

**Methods:**

In this case-control study, 89 samples (including 45 carcinoma and 44 non-carcinoma samples) were examined. The carcinomatous samples were selected by the census method and others were selected with random method. The data were obtained from histopathologic diagnosis, immunohistochemistry (negative, positive and intensity of immunoreactivity), age, history of diabetes, and hypertension. Ob-R expression was compared in the studied groups using Chi-square, Fisher tests and Multivariate logistic regression analysis. In all tests the level of significance was set at 0.05. The SPSS 26 was used for data analysis.

**Results:**

The frequency of high levels of leptin receptors in the patients with endometrial carcinoma was significantly higher compared to the control group (57.8% vs. 2.3%) (*P*<0.05). Adjusting the effects of age, history of diabetes mellitus (DM) and hypertension (HTN) revealed that the positive-receptor group had 37.75 (95% CI; 5.18-275.04) odds of having endometrial carcinoma (*P*<0.001).

**Conclusion:**

The leptin receptor may be a risk factor for the endometrial carcinoma among women tested in Kashan. Based on these results, leptin receptor might be considered as a potential biomarker for screening the endometrial carcinoma or targeting the therapeutic purposes.

## Introduction

Endometrial carcinoma is one of the most common malignancies in women in developed countries (1) and the fourth malignancy in Iranian women after breast, lung, and colorectal cancer. In terms of pathogenesis, there are two types; the most common type, endometrioid carcinoma, is estrogen-dependent and occurs in the context of endometrial hyperplasia and has well-known factors such as obesity, hypertension (HTN), diabetes mellitus (DM), ovulation failure, and long-term estrogen use. The other one is serous carcinoma that is identified in the elderly women with no known background. It is independent of estrogen with bad prognosis (2). 

The most prevalent sign of this cancer is abnormal uterine bleeding (AUB) particularly in women above the age 45 years. About 10%of AUB after menopause is due to endometrial carcinoma (3). A 5-year survival rate of 100% may be predicted for a low degree endometrial carcinoma with less than 50% myometrial invasion (4). 

Immunologic biomarkers such as CK7, CK8, Vimentin, CK19, CK18, and PAX8 have been used to diagnose this disease, however, none of these factors have shown sufficient sensitivity or specificity for the diagnostic purposes (1); the gold standard diagnostic method is endometrial biopsy (5) as no screening test has been introduced up to date (6). 

Leptin is one of the markers used to diagnose endometrial cancer. This is a 16 KD adiponectin hormone produced by Ob gene of the adipose tissue. It has roles in regulating food intake, reproduction, body weight, hematopoiesis, angiogenesis, bone formation, and wound healing. It has a potential role in growth and is a mitogen factor in normal endometrium and endometrial cancer as well (7-11). 

The influence of leptin on proliferation and invasion of endometrial cancer cells depends on the dose and the rate of leptin receptor expression (Ob-R) (12-14). Leptin receptors are often located in the cell membrane or cytoplasm of normal and tumoral endometrial cells (15). 

Considering these evidence, it seems that this biomarker may play a significant role in the disease diagnosis. Therefore, this research was designed to compare the leptin receptor expression in the endometrial carcinoma versus the non-cancer lesions of endometrium and normal tissue.

## Material and Methods

This case-control research was done on 45 endometrial carcinoma samples as the case group and 44 non-endometrial carcinoma samples including proliferative and secretory phases and non-atypical hyperplastic cases as the control group. The carcinomatous and other samples were selected by the census method and random method, respectively. We had no matching between the two groups due to insufficient samples in the groups. These two groups were compared for the leptin receptor positivity with Chi-square and Fisher exact tests. The slides and blocks of dilatation and curettage (D&C) and hysterectomy samples dating from the years 2012 to 2020 were collected from the archive of the Pathology Laboratory of Shahid Beheshti Hospital. First, by microscopic examination of the slides by a pathologist, the blocks with optimal quality for the immunohistochemistry (IHC) staining of Ob-R were selected and other information such as age, history of DM and HTN were extracted from the patients' file.

**Immunohistochemical Staining**

Immunohistochemical staining was accomplished using antibodies that recognize the target protein. Since the antibodies are highly specific, they bind only to the protein of interest in the tissue section. The antibody-antigen interaction was then visualized using chromogenic detection, in which an enzyme conjugated to the antibody cleaves a substrate to produce a colored precipitate at the location of the protein. 

IHC-P refers to the staining of tissues that have been fixed (usually in neutral buffered formalin) and then embedded in paraffin before being sectioned. 

Tissue sections with the thickness of 2-3 µM were prepared by microtome from the selected paraffin blocks. For the IHC staining, Anti-Leptin Receptor Antibody kit (ab216610) from Abcam, UK was used. 

First, the slides were deparaffinized and rehydrated. They were washed as following: Xylene** (**2 x 3 min), Xylene and 100% ethanol (1:1): 3 min, descending alcohol (100, 95, 70, 50 degree): 3 min, then rinsed by cold tap water. For removing antigen mask, the slides were placed in Tris-EDTA Buffer (pH=9) and microwave at 98°C for 20 min. Then, blocking peroxidase was poured over the slides and placed in dark for 10 min, then, moved to the room temperature for 10 min. Then, washed with distilled water and TBS buffer. The primary anti**-**Leptin antibody at 1:100 dilution was poured on the slides. The slides were incubated for 1 h at room temperature and washed in PBS buffer. The secondary antibody was added to the sections in 2 steps and incubated for 20 min each. The sections were placed in diluted chromogen solution with special buffer for 5 min and then washed with water. Staining background with hematoxylin was performed for 10 sec; then, it was washed in running water. Dehydration of tissues was done by using descending alcohol (100, 95, 70 degree). At the end, the slides were placed in Xylol solvent, mounted, and examined by the Olympus cx23 microscope. 

**IHC Scoring**

Based on the antibody Kit recommendation, placental trophoblastic tissue was used as the positive control. After microscopic examination, the samples were classified into positive and negative categories. The positive samples were graded based on the intensity of staining from +1 to +3. They were considered as follow: Samples without staining or weak staining in less than 10% of cells (score 0, negative), samples with weak to moderate staining in 10 to 29% of cells (score 1, positive), samples with moderate to intense staining in 30 to 49% of cells (score 2, positive), and samples with intense staining in more than or equal to 50% of cells (score 3, positive).

**Statistical Analysis**

The obtained data related to each sample including histopathologic diagnosis, immunohistochemistry results (negative, positive and intensity of immunoreactivity), and data related to the patients’ age, history of diabetes and hypertension were analyzed by SPSS version 26 (SPSS Inc., Chicago, Ill., USA). 

In order to calculate the sample size, the results of previous studies (reference) and observation of Ob-R expression in the two non-carcinoma (34±98) and carcinoma groups (68±177) and a difference of d=30 digital score with 80% power and 95% confidence interval (CI), the sample size was calculated 45 patients in each group. The formula of sample size is as follow:

$n=\frac{\left(Z_{1-\frac{\alpha }{2}}+Z_{1-\beta }{)}^{2}\times {(S}_{1}^{2}+S_{2}^{2}\right)}{(\overset{\text{®}}{X_{1}}-\overset{\text{®}}{X_{2}}{)}^{2}}$

Z1-a/2=1.96

Z1-B=0.84

S1=standard deviation of group 1

S2=standard deviation of group 2

X1=mean of group 1

X2=mean of group 2

Due to the lack of access to the information about weight and BMI and the incompleteness of the patients’ files these items were not included in the study. Leptin receptor (Ob-R) positivity was compared with Chi-square and Fisher tests. Also, multivariate logistic regression analysis was used to determine the association between leptin receptor and endometrial cancer by removing the confounding variables of age, history of diabetes mellitus and hypertension. 

The reason for selecting this method was the limited number of cases available in sub-groups with different levels of leptin receptor expression. Thus, by merging the leptin positive groups, independent variable was divided into positive and negative receptors. In all tests the level of significance was considered at P-value<0.05. 

**Fig. 1:**
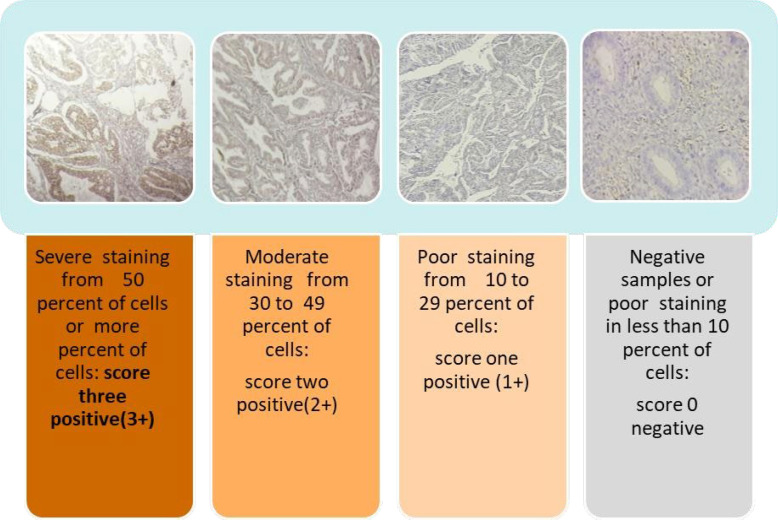
Intensity of the leptin receptor staining in the studied endometrial tissues

Figure 1 shows the results of leptin immunohistochemical staining according to the intensity of staining. 

## Results

This study was performed on 100 samples archived in the Pathology Laboratory of Shahid Beheshti Hospital, Kashan University of Medical Sciences. All the examined samples were from 2012 to 2020. Of all the selected samples, 11 samples were excluded from the study due to the technical staining problems, but 89 samples were examined.

**Table 1:** The frequency of DM and HTN in the study groups

History		Study group		P-value
		Cancer (%)	Non-cancer (%)	
DM	Yes	17 (73.9)	6(26.1)	<0.01
	No	28 (42.4)	38 (57.6)	
HTN	Yes	12 (85.7)	2 (14.3)	<0.01
	No	33 (44.0)	42 (56.0)	

In immunohistochemical staining, leptin receptor expressions in the case and control groups were 57.8 and 2.3%, respectively, which was statistically highly significant (*P*<0.001). In addition, the rate of expression in the case group was significantly higher than the control group (*P*<0.001) (Table 2). 

**Table 2:** Comparing the intensity of leptin receptor expression in endometrial cancer versus the control group

Group Expression rate	Control		Endometrial carcinoma		Fisher’s exact test
	Number	Percent	Number	Percent	< 0.001
Negative	14	31.8	0	0	
Low	15	34.1	8	17.8	
Moderate	14	31.8	11	24.4	
High	1	2.3	26	57.8	
Total	44	100	45	100	

In order to eliminate the effects of confounding variables, the samples were divided into positive and negative groups in terms of gene expression. The association between gene expression and carcinoma incidence was investigated using multivariate logistic regression analysis. 

The results of the analysis showed that among the studied variables only age and receptor expression had a significant association with cancer. With an increase of one year of age, the chance of developing cancer increased by 1.18 (95% CI; 1.09- 1.27) (*P*<0.001). The chance of cancer in cases with positive expression (95% CI; 5.18-275.04) was 37.75 times higher than in cases with negative receptor (*P*<0.001). Other details are presented in Table 3. 

**Table 3:** The results of multivariate logistic regression analysis on the association between leptin receptor expression and endometrial carcinoma

Variable	Odds ratio	S.E.	Z	P-value	95% Confidence Interval	
Age	1.18	0.04	4.47	<0.001	1.09	1.27
Diabetes Mellitus	1.62	1.17	0.67	0.50	0.39	6.69
Hypertension	3.21	2.94	1.27	0.20	0.53	19.37
Expression	37.75	38.25	3.58	<0.001	5.18	275.04
Constant	4.15e-06	0.000	-4.99	<0.001	3.19e-0.08	0.0005

## Discussion

In this study, the expression of leptin receptor was examined in the endometrial carcinoma and non-carcinoma samples. Based on the results, the rate of leptin receptor expression in carcinoma patients was significantly higher than the non-carcinoma samples. This finding was similar to the results of research reported by Carino *et al.,* (2008) who showed that the rate of leptin receptor expression in the patients with endometrial carcinoma was higher than the benign endometrial cells (14). 

Also, regarding the association between leptin expression and clinical-pathological parameters in endometrial carcinoma, Khabaz *et al.,* (2016) confirmed the diagnostic and prognostic values of leptin in endometrial carcinoma and suggested that this molecule is involved in the development of endometrial tumor. Leptin may be a valuable marker for the prediction of tissue type, stage, recurrence, and poor prognosis (16, 17). These results are consistent with our study outcome. 

In regard to the association of leptin receptor and endometrial carcinoma, Sharma, and associates (2006) found that leptin treatment resulted in the increase of type-I Ishikawa/ECC1 endometrial carcinoma cells that respond to the hormone (18). 

This finding has been investigated in more detail. In the study of Gong* et al., *(2007), Ishikawa cells were exposed to leptin at different concentrations and different time points. They observed that leptin stimulates the proliferation of Ishikawa cells (19). Also, Wu X* et al., *(2012) found that the exposure to leptin stimulates the proliferation and invasion of SPEC-2 cells (type-II endometrial carcinoma cell line) (20). Carino* et al., *(2008) showed that leptin is involved in regulating the vascular endothelial growth factor (VEGF), IL-1ß, leukemia inhibitor factor, and the receptors of VEGFR2, type*-1R type *I (IL-1R tI) and LIFR in a dose-dependent manner (14).

The effect of leptin on pre angiogenesis molecules in malignant cells is more than the benign cells. Leptin causes higher secretion level in VEGF/VEGFR2 and LIF in carcinoma than benign cells (14). Regarding the role of leptin in programmed cell death, the results of a study on endometrial carcinoma conducted by Zhou Chai (2015) showed leptin decreases the apoptosis in Ishikawa/ECC1 cells. Therefore, it is conceivable that the presence of leptin in tumor micro-environment can play a significant role on the biological mechanisms such as cell growth, invasion, and escaping apoptosis. The previous studies have also shown that leptin has the potential to stimulate cell growth, invasion, and angiogenesis through activating different cell signaling pathways that may cause endometrial carcinoma progression. Despite all these facts, it seems that the potential mechanisms involved in the association between the endometrial carcinoma and leptin are complicated and require further research in future (21, 22). Research findings by Cymbaluk* et al., *(2018) demonstrated that high level of serum leptin is associated with an increase in the endometrial carcinoma (23-25). 

In Zhou *et al.,* study (2015), the expression of Ob-R receptors in poorly and moderately differentiated endometrial carcinoma cells was higher than in well differentiated types, and a higher incidence of the receptor was seen in the clinical stages two and three compared to the stage one (*P*=0.012). It seems that the high expression of Ob-R receptor facilitates the progression of endometrial carcinoma (26), which confirms the results of our study. 

The study conducted by Wang *et al.,* (2006) also showed that the expression of Ob-R in poorly differentiated endometrial carcinoma was much higher than in well differentiated endometrial carcinoma, and leptin can inhibit apoptosis in endometrial carcinoma cells by increasing the activity of the NIK/IKK signaling pathway (27). 

The results of Mendez and Zavala study (2017) on 6 groups (proliferative endometrium, secretory endometrium, non-atypical hyperplasia, atypical hyperplasia, endometrioid and non-endometrioid carcinoma) showed leptin receptor down-regulation during the progression of endometrial carcinoma and its decrement in tumoral tissues compared to the normal tissue (28). These results were not consistent with our study results. 

Also, in the study of Ozler (2011), no difference was noticed in the intensity of staining among proliferative, secretory, and hyperplastic endometrium (*P*<0.05), therefore, it was concluded that leptin does not play a role in the hyperplastic change of the endometrium (29). This result was contrary to our results. 

One of the interesting findings of the present research was the high frequency of diabetic patients suffering from endometrial carcinoma that were significantly higher than the control group (37.8% with a ratio of 13.6) (*P*<0.009). This finding was in agreement with the findings of research reported by Byrne (2020) (30). 

One of the limitations of our study was the small sample size; therefore, we could not investigate different degrees of endometrial carcinoma. Also, due to the limitation of financial resources, it was not possible to include the atypical hyperplasia samples examination. In addition, due to the lack of access to the patients, the leptin serum level of the patients was not checked. It is suggested that future studies are carried out with a larger sample size, including atypical hyperplasia and endometrial carcinoma according to the degree of differentiation. Also, the serum level of leptin should be investigated and the diagnostic accuracy of leptin receptor in the endometrial cancer should be determined based on the R.O.C (Receiver Operating Characteristics) curve and the cut point.

## Conclusion

The leptin receptor may be a risk factor for the endometrial carcinoma, which was found among the evaluated women in Kashan. Based on these results, leptin receptor should be considered as a potential biomarker for the endometrial carcinoma screening or targeting the therapeutic purposes.

## Conflict of Interest

The authors declare no conflicts of interest.
